# A Bayesian Analysis for Identifying DNA Copy Number Variations Using a Compound Poisson Process

**DOI:** 10.1155/2010/268513

**Published:** 2010-08-17

**Authors:** Jie Chen, Ayten Yiğiter, Yu-Ping Wang, Hong-Wen Deng

**Affiliations:** 1 Department of Mathematics and Statistics, University of Missouri-Kansas City, Kansas City, MO 64110, USA; 2 Department of Statistics, Hacettepe University, 06800Beytepe-Ankara, Turkey; 3 Biomedical Engineering Department, Tulane University, New Orleans, LA 70118, USA; 4 Departments of Orthopedic Surgery and Basic Medical Sciences, School of Medicine, University of Missouri-Kansas City, Kansas City, MO, 64108, USA

## Abstract

To study chromosomal aberrations that may lead to cancer formation or genetic diseases, the array-based Comparative Genomic Hybridization (aCGH) technique is often used for detecting DNA copy number variants (CNVs). Various methods have been developed for gaining CNVs information based on aCGH data. However, most of these methods make use of the log-intensity ratios in aCGH data without taking advantage of other information such as the DNA probe (e.g., biomarker) positions/distances contained in the data. Motivated by the specific features of aCGH data, we developed a novel method that takes into account the estimation of a change point or locus of the CNV in aCGH data with its associated biomarker position on the chromosome using a compound Poisson process. We used a Bayesian approach to derive the posterior probability for the estimation of the CNV locus. To detect loci of multiple CNVs in the data, a sliding window process combined with our derived Bayesian posterior probability was proposed. To evaluate the performance of the method in the estimation of the CNV locus, we first performed simulation studies. Finally, we applied our approach to real data from aCGH experiments, demonstrating its applicability.

## 1. Introduction

Cancer progression, tumor formations, and many genetic diseases are related to aberrations in some chromosomal regions. Chromosomal aberrations are often reflected in DNA copy number changes, also known as copy number variations (CNVs) [1]. To study such chromosomal aberrations, experiments are often conducted based on tumor samples from a cell-line-using technologies such as aCGH or SNP arrays. For instance, in aCGH experiments, a DNA test sample and a diploid reference sample are first fluorescently labeled by Cy3 and Cy5. Then, the samples are mixed and hybridized to the microarray. Finally, the image intensities from the test and reference samples can be obtained for all DNA probes (bio-markers) along the chromosome [2, 3]. The log-base-2 ratios of the test and reference intensities, usually denoted as , are used to generate an aCGH profile [4]. To reduce noise, the Gaussian-smoothed profile is often used. With an appropriate normalization process,  is viewed as a Gaussian distribution of mean 0 and variance  [4, 5]. The deviation from mean 0 and variance  in  data may indicate a copy number change. Therefore, detecting DNA copy number changes becomes the problem of how to identify significant parameter changes occurred in the sequence of  observations.

There are a number of computational and statistical methods developed for the detection of CNVs based on aCGH data and SNP data. Examples include a finite Gaussian mixture model [6], pair wise *t*-tests [7], adaptive weights smoothing [8], circular binary segmentation (CBS) [4], hidden Markov modeling (HMM) [9], maximum likelihood estimation [10], and many others. A comparison between several of these methods for the analysis of aCGH data was given by Lai et al. [11]. There are continued efforts on developing methods for accurate detection of CNVs. Nannya et al. [12] developed a robust algorithm for copy number analysis of the human genome using high-density oligonucleotide microarrays. Price et al. [13] adapted the Smith-Waterman dynamic programming algorithm to provide a sensitive and robust approach (SW-ARRAY). More recently, Shah et al. [14] proposed a simple modification to the hidden Markov model (HMM) to make it be robust to outliers in aCGH data. Yu et al. [15] developed an edge detection algorithm for copy number analysis in SNP data. An algorithm called reversible jump aCGH (RJaCGH) for identifying copy number alterations was introduced in Rueda and Díaz-Uriarte [16]. This RJaCGH algorithm is based on a nonhomogeneous HMM fitted by reversible jump MCMC using Bayesian approach. Pique-Regi et al. [17] proposed to use piecewise constant (PWC) vectors to represent genome copy number and used sparse Bayesian learning (SBL) to detect copy number alterations breakpoints. Rancoita et al. [18] provided an improved Bayesian regression method for data that are noisy observations of a piecewise constant function and used this method for CNV analysis. We have formulated the problem as a statistical change-point detection [19] and proposed a mean and variance change-point model (MVCM), which brought significant improvement over many existing methods such as the CBS proposed by Olshen et al. [4].

The above-mentioned algorithms, however, have not taken advantage of other information such as the positions of the DNA probes or biomarkers along the chromosome. Recently, many researchers have begun to consider variations in the distance between biomarkers, gene density, and genomic features in the process of identifying increased or decreased chromosomal region of gene expression [5]. Several notable methods emerged along this line and we list a few of them here. Levin et al. [5] developed a scan statistics for detecting spatial clusters of genes on a chromosome based on gene positions and gene expression data modeled by a compound Poisson process on the basis of two independent simple Poisson processes. Daruwala et al. [20] developed a statistical algorithm for the detection of genomic aberrations in human cancer cell lines, where the location of aberrations in the copy numbers was modeled by a Poisson process. They distinguished genes as "regular" and "deviated", where the regular genes refer to those that have not been affected by chromosomal aberrations while the deviated genes are those whose log-transformed expression follows a Gaussian distribution with unknown mean and variance [20]. Sun et al. [21] developed a SNP association scan statistic similar to that of Levin et al. [5] using a compound Poisson process, which considers the complex distribution of genome variations in chromosomal regions with significant clusters of SNP associations.

Improvements have been made with the above more sophisticated modeling of the aCGH using both the log-intensity ratios and biomarker positions. The computation involved in this type of modeling is usually demanding and further improvement is needed. Motivated by these existing works, we propose to use a compound Poisson process approach to model the genomic features in identifying chromosomal aberrations. We use a Bayesian approach to determine an aberration (or a change-point) in the aCGH profile modeled by a compound Poisson process. In our model, the occurrences of the biomarkers are modeled by a homogeneous Poisson process and the aCGH is modeled by a Gaussian distribution. This novel method is able to identify the aberration corresponding to the CNVs with associated distance between biomarkers on the chromosome. The proposed method is inspired by the scan statistic [5, 21], which is widely used for identifying chromosomal aberrations. However, our method differs from the work of Levin et al. [5] in that our method uses a statistical change-point model with a compound Poisson process for the identification of CNVs.

## 2. Methods

### 2.1. Modeling aCGH Data Using a Compound Poisson Change Point Model

To describe our approach, we first describe a change-point model for a compound Poisson process in terms of the normalized log ratio  and the biomarker distances along a chromosome, where  and  and  are the intensities of the test and reference samples at locus  on the chromosome (or genome). Based on probability distribution theories and characteristics of the hybridization process of aCGH technique, the occurrence of the biomarkers on the chromosome can be modeled by a homogeneous Poisson process. Similarly to the notations adopted in Levin et al. [5] and Sun et al. [21], we denote  as a simple (homogeneous) Poisson process with the rate parameter , where  is the number of biomarkers occurring over a given base pair length  and  is the occurrence rate of biomarkers over a distance of t base pairs along the chromosome. Let  represent the positions of the biomarkers on a chromosome and(1)

represent the distance between the th biomarker and the th biomarker. Since  is a homogeneous Poisson process, according to probability distribution theories, s are independent and identically distributed (iid) with exponential variables with parameter ; furthermore, s are gamma distributed with rate parameter  and scale parameter i, and the probability density function as follows:(2)

where  is the gamma function, and  for a positive integer .

Note the fact that the distances s are iid exponential random variables can be used to verify the assumption on the occurrence of  being a simple (homogeneous) Poisson process.

Assume that the given interval with base pair length  is divided by the nonoverlapping subintervals with lengths . Then, the sequence of the log intensity ratio, , corresponding to each subinterval can be denoted as  and clearly(3)

Given that  is a homogeneous Poisson process and  follow independent Gaussian (normal) distributions [5] with mean  and variance , ,  is then defined by a compound Poisson process, where the ,  are independently and normally distributed with mean  and variance , respectively. The number, , of biomarkers in each subinterval of length  is distributed as a Poisson distribution with parameter  (where  represents the occurrence rate of biomarkers or SNPs corresponding to subinterval ) for .

The problem is if there is an aberration (increase or decrease) in the sequence  at an unknown locus  with base pair length . In statistical change-point modeling theory, this is to know if there is a change in the parameters of the distribution of the independent sequence of  at an unknown point  (change point) contained in the interval with length . Specifically, the change point model in the compound Poisson process can be formulated as (4)

where ,  and  are unknown means,  is unknown variance of the normal distribution, and , , and  are unknown mean rates of biomarker occurrences in each subinterval. The goal of the study becomes to estimate the value of .

**Figure 1:**
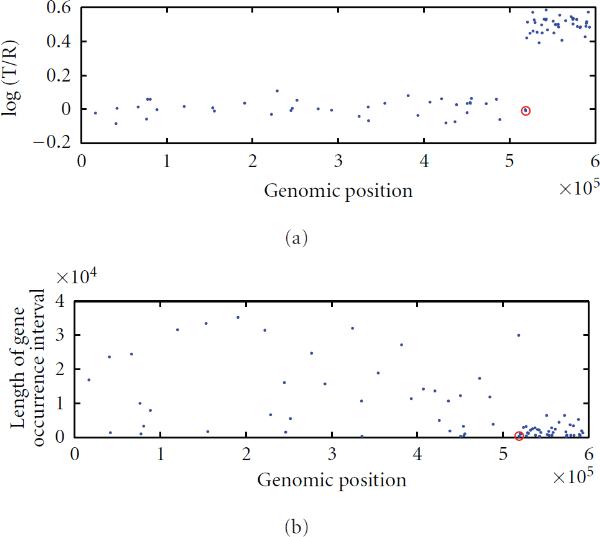
**Simulated compound Poisson process data with one change: The upper panel is a plot of the simulated log ratio intensities (normally distributed) against the genomic positions, and the lower panel is a plot of the interval length against the corresponding genomic positions (distributed with Poisson)**.

For illustration purpose, in the following Figure 1, we provide a scatter plot that represents a change in a sequence of data simulated from a compound Poisson process described above.

### 2.2. A Bayesian Analysis for Locating the Change Point

The change-point model in the compound Poisson process described above can be viewed as a hypothesis testing problem. It tests the null hypothesis, , of no change in the parameters of the sequence of random variables  in subintervals with length (5)

versus the alternative hypothesis(7)

The alternative hypothesis (7) above defines a change-point model. For this model, we propose a Bayesian approach for the estimate of . Due to the requirement of occurrence in an interval, we only consider the search of the change when  is between 2 and . We will obtain the posterior distribution of  in the sequel. We first assume that the prior distribution of  is taken as an noninformative prior(8)

The following joint prior distribution is given for ,  and (9)

and for the common variance , the prior distribution is taken as(10)

Under those assumptions, the likelihood function of  can be written as (11)

The joint posterior distribution of the parameters , , ,  and  is then obtained as(12)

Integrating (12) above with respect to , ,  and , we found that the marginal posterior distribution of the interval  that included the change point is proportional to(13)

for , where the constants A, B, and C in (13) are obtained as (14)

The probability  in (13) is computed from the Poisson distribution with parameter  for  according to the Poisson model under the alternative hypothesis  (7), or namely(15)

In order to compute the probability given by (15), the occurrence rates , , and  can be estimated with the maximum likelihood estimator (MLE), , , and , in the subintervals of lengths , , and , respectively. These MLEs are easily obtained as(16)

With these MLEs, (15) becomes(17)

Therefore, with the Poisson probabilities given by (17),  in (13) can be rewritten as(18)

Finally, the marginal posterior distribution of the locus  is obtained as(19)

where  is given in (18). The estimate of the change locus  is then given by  such that the posterior distribution (19) attains its maximum at , that is, (20)

Based on the above theoretical results, we provide the computational implementation of our approach in the next subsection.

### 2.3. Computational Implementation of the Bayesian Approach

To implement our above Bayesian approach to real data, it is necessary to define the number, , of subintervals at first. Our numerical experiments show that the number, , of subintervals can be chosen such that each subinterval includes at least one observation (log ratio ) and at most 300 observations. The lengths, , ,, and , of the subintervals can be chosen equally (in this case, the numbers of biomarkers contained in each subinterval are not equal). An easier option of choosing the length, , for subinterval  is to have each subinterval to contain the same number of observations. From a practical point of view, the number of subintervals, , and the size of each subinterval can also be defined by users according to their prior knowledge about their data.

Although our approach was given for the single change-point model in compound Poisson process, it can be easily extended to the multiple change points (or aberrations) by using a sliding window approach [21, 22]. Sun et al. [21] have taken the sliding window sizes as 3 to 10 consecutive markers in their application. Our numerical experiments suggest that the sliding window of sizes ranging from 12 to 35 subintervals should be effective in searching for multiple changes in the aCGH data based on our proposed Bayesian approach. To avoid intermediate edge problems within each window, the two adjacent windows have to overlap. Many of such issues were also discussed in [22]. For the searching of multiple change points with the sliding window approach, a practical question is how to set the threshold value for the maximum posterior probabilities associated with all windows. In our application, we used the heuristic threshold of 0.5 (which is popular in probability sense) for the maximum posterior probabilities.

As a summary of our method, we give the following steps to implement our proposed Bayesian approach to the compound Poisson change-point model (Bayesian-CPCM).

(1) If it is known that a chromosome has potentially one aberration region, calculate the posterior probability (19) and identify the locus  according to (20).

(2) If there are multiple aberration regions on a chromosome or genome, choose a total of  sliding windows with sizes ranging from 12 to 35 such that *each window contains exactly one potential aberration*. Denote these  windows by ,,...,, where  equals the total number of observations on the chromosome.

(3) For window , determine the number of subintervals  with lengths ,,.

(4) Count the number of biomarkers, , in each subinterval with length , .

(5) Compute the posterior probabilities for  using (19), find the maximum of the posterior probability distribution. If the maximum posterior probability is larger than 0.5 (or larger than a selected threshold according to practice) at , then identify  according to (20).

(6) Convert the identified change position  into the actual biomarker position , and declare  as the position on the chromosome at which the CNV has changed.

(7) Repeat steps 3–6 above for , where J is determined by the final window size and the final window size is determined at the value for which the posterior probabilities stabilize. 

The Matlab code of the Bayesian-CPCM approach has been written and is available upon readers' request.

## 3. Results

### 3.1. Simulation Results

The proposed method provides a theoretic framework of detecting CNVs using both biomarker positions and log-intensity ratios. Since there is no suitable metric that can be used to compare the proposed approach with all existing algorithms, we carried simulation studies based on a commonly used approach for evaluating the estimation of a change point. We simulated sequences as independent normal distributions with moderate sample size  (the sequence size) of 12, 20, 32, 40, 80, and 120 for the scenarios of the changes being located at the front (the th observation), at the center (the th observation), and at the end (the th observation) of the respective sequence. For the choices of the mean and variance parameters before and after the change location, we consider the specific features of the real aCGH data. Using data from the fibroblast cell lines as benchmarks, we observed that the segments before and after a detected change point mostly have mean difference ranging from  .36 to  .7 (or larger), and a standard deviation difference ranging mostly from  .05 to  .2. We, therefore, investigated the cases when the mean and the standard deviation are within the above-mentioned ranges. Due to the page limit of the paper, we only report part of the simulation results in Table 1. In Table 1,  denotes the true change location;  is the estimated change location according to (20);  represents the relative frequency that the estimated location  equals to the true location ; and  is the mean squared error of the location estimator. Each simulation is carried out 1,000 times.

**Table 1:** Simulation results

	When				When			
								
	3	2.8870	0.8210	0.4034	3	2.8960	0.8630	0.2903
12	6	5.9710	0.9040	0.3774	6	5.9510	0.9070	0.4635
	9	8.7930	0.8560	1.6906	9	8.9130	0.8940	0.8038

	5	5.0010	0.9800	0.0230	5	5.0050	0.9910	0.0150
20	10	10.0180	0.9800	0.0200	10	10.0110	0.9850	0.0150
	15	15.0090	0.9800	0.0310	15	15.0130	0.9810	0.0190

	8	8.0070	0.9930	0.0070	8	8.0040	0.9960	0.0040
32	16	16.0020	0.9900	0.0100	16	16.0000	0.9980	0.0020
	24	24.0020	0.9960	0.0040	24	23.9980	0.9980	0.0020

	10	10.0020	0.9980	0.0020	10	10.0030	0.9970	0.0000
40	20	20.0040	0.9960	0.0040	20	20.010	0.9990	0.0010
	30	30.0000	1.0000	0.0040	30	30.0010	0.9990	0.0010

	20	20.000	1.0000	0.0000	20	20.0000	1.0000	0.0000
80	40	40.0000	1.0000	0.0000	40	40.0000	1.0000	0.0000
	60	60.0000	1.0000	0.0000	60	60.0000	1.0000	0.0000

	30	30.0030	0.9970	0.0030	30	30.0000	1.0000	0.0000
120	60	60.0000	1.0000	0.0000	60	60.0000	1.0000	0.0000
	90	90.0000	1.0000	0.0000	90	90.0000	1.0000	0.0000

In this table, , , , , , and .

The simulation results given in Table 1 indicate that the derived posterior probability (19) can identify changes in the front, the center and the end of the sequence, respectively, with very high certainty—at least 97% for sample sizes of 20 or larger. The average of the estimated locations is remarkably close to the true change locus with very small MSE. The proposed method can be confidently applied to the identification of DNA copy number changes.

### 3.2. Applications to aCGH Datasets on 9 Fibroblast Cell lines

Several aCGH experiments were performed on 15 fibroblast cell lines and the normalized averages of the  (based on triplicate) along positions on each chromosome were available at the following website [23]: http://www.nature.com/ng/journal/v29/n3/full/ng754.html. For the missing values in the log ratio values, we imputed 0 into the original data. The DNA copy number alterations in each of the 15 fibroblast cell lines were verified by karyotyping [23]. Therefore, these 15 fibroblast cell lines aCGH datasets can be used as benchmark datasets to test our methods.

For the 9 fibroblast cell lines analyzed in many followup papers of [23], we also used our posterior probabilities (19) to locate the locus (or loci) on those chromosomes where the alterations had been identified. It turned out that our method can identify the locus (or loci) of the DNA copy number alterations that are exactly corresponding to the karyotyping results [23]. The CNVs found by our proposed Bayesian approach (with sliding windows when appropriate) are summarized in the following Tables 2 and 3.

**Table 2:** Results of the Bayesian approach on chromosomes with one change identified

Cell line	Chromosome	(kb)	
GM01535	chromosome 5	176824	.5237
GM01750	chromosome 9	26000	.9666
GM01750	chromosome 14	11545	.7867
GM03563	chromosome 3	10524	.8808
GM03563	chromosome 9	2646	1.000
GM07081	chromosome 7	57971	.6390
GM13330	chromosome 1	156276	.9994
GM13330	chromosome 4	173943	.9999

The posterior probability shown is the maximum posterior probability for the chromosome.

**Table 3:** Results of the Bayesian approach on chromosomes with two changes identified

Cell line	Chromosome	(kb)		Window size
GM01524	chromosome 6	74205, 145965	.9501, .7411	17
GM03134	chromosome 8	99764, 146000	.9397, 9602	20
GM05296	chromosome 10	64187, 110412	.7229, .8955	30
GM05296	chromosome 11	34420, 43357	.8496, .9852	18
GM13031	chromosome 17	50231, 58122	.9434, .7701	20

The posterior probability shown is the maximum posterior probability for the chromosome at the respective loci.

**Figure 2:**
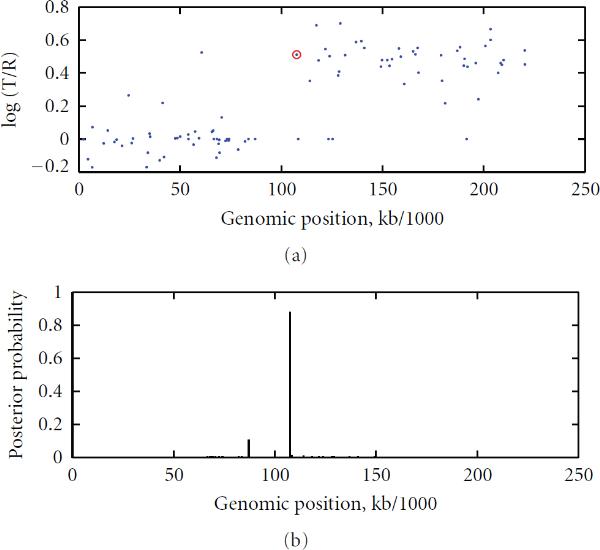
**Chromosome 3 of GM03563 **[23] **with identified change locus and the posterior probability distribution: A red circle indicates a significant DNA copy number change point such that the segment before this red circle (inclusive of the red circle) is different from the successor segment after the red circle (exclusive of the red circle)**

**Figure 3:**
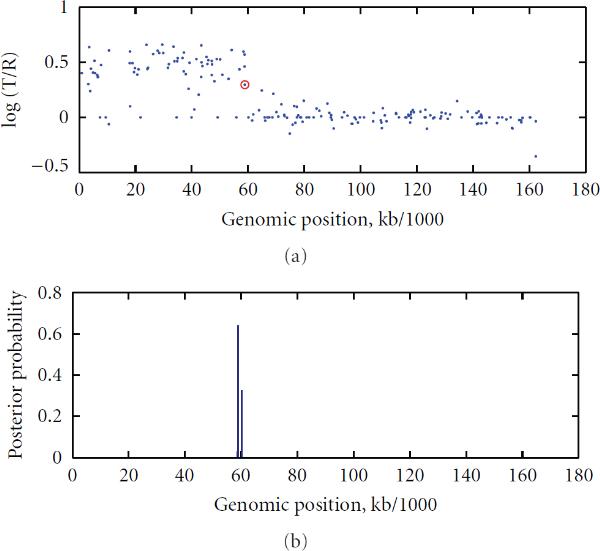
**Chromosome 7 of GM07081 **[23] **with identified change locus and the posterior probability distribution: A red circle indicates a significant DNA copy number change point such that the segment before this red circle (inclusive of the red circle) is different from the successor segment after the red circle (exclusive of the red circle)**.

According to the posterior probability (19), we found that there was one copy number change on chromosome 5 of the cell line GM01535, chromosomes 9 and 14 of the cell line GM01750, chromosomes 3 and 9 of the cell line GM03563, chromosome 7 of the cell line GM07081, and chromosomes 1 and 4 of the cell line GM13330. No false positives were found on these chromosomes with the threshold of 0.5 for the maximum posterior probability (20). These findings are consistent with the karyotyping result of Snijders et al. [23]. In Figures 2 and 3, we give the scatter plots of the aCGH data of Chromosome 3 of GM03563, and of Chromosome 7 of GM07081, along with their respective posterior probability distributions. The peak posterior indicated a change at that genomic locus. The beginning point after which the corresponding log ratio values are increased is circled as red.

**Figure 4:**
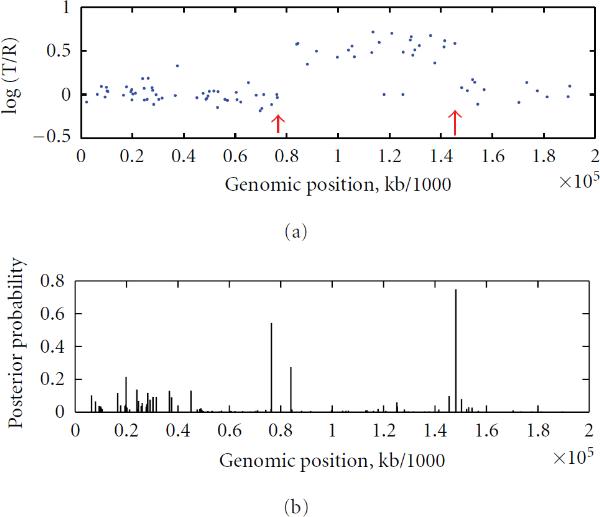
**Chromosome 6 of GM01524 **[23] **with identified change loci (indicated by red arrows) and the posterior probability distributions with a window size of 20**

**Figure 5:**
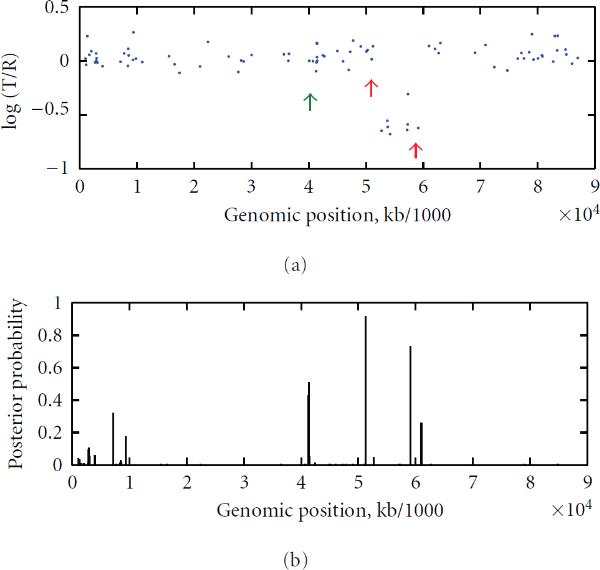
**Chromosome 17 of GM13031 **[23] **with identified change loci (indicated by red arrows, while the green arrow indicates a false positive) and the posterior probability distributions with a window size of 20**.

Our posterior probability function of (20) combined with the sliding window approach signals two or more possible copy number changes on chromosome 6 of GM01524, chromosome 8 of GM03134, chromosomes 10 and 11 of GM05296, and chromosome 17 of GM13031. These results were given in Table 2. Figures 4 and 5 give the findings on Chromosome 6 of GM01524 and Chromosome 17 of GM13031, respectively, with a sliding window approach used. These findings are again consistent with the karyotyping result of [23].

### 3.3. Comparison of the Performances of the Proposed Bayesian-CPCM with CBS on the Fibroblast Cell-Lines Datasets

There are many approaches (computational or statistical) now available for analyzing aCGH data in the relative literature. But many of those approaches, especially CBS [4], have targeted on modeling the log ratio intensity in aCGH data. Now, in this paper, we have used a new concept to model both the gene position and the log ratio intensity in aCGH data. That is, the most distinct feature of the proposed Bayesian-CPCM approach, among other existing methods in the literature, is its usage of the information of the gene positions (hence gene distances) and the log ratio intensities in the model.

Although there is no suitable metric that can be used to compare all the existing methods for CNV data analysis, we used the specificity and sensitivity as comparison metric to evaluate the performance of our proposed method with one of the most popularly used CBS method. The comparison results are given in the following Table 4. In Table 4, "Yes" means the change was found by the specific method (CBS or Bayesian-CPCM) for the known alteration verified by spectral karyotyping in Snijders et al. [23] on the specific chromosome in the cell line at the given  level (for the case of using CBS or MVCM) or with maximum posterior probability larger than 0.5 (for the case of using Bayesian-CPCM), "No" means the change was not found by a specific method, but was identified by spectral karyotyping; and "Number of false positives" gives the number of changes found by the specific method for a cell line while there were no known alterations actually found by spectral karyotyping [4, 23].

**Table 4:** Comparison of the changes found using CBS and the proposed Bayesian-CPCM on the nine fibroblast cell lines

Cell line/chromosome	CBS		Bayesian-CPCM approach
			
GM01524/6	Yes	Yes	Yes
Number of false positives	6	2	0
Specificity	72.7%	90.9%	100%
Sensitivity	100%	100%	100%

GM01535/5	Yes	Yes	Yes
GM01535/12	No	No	No
Number of false positives	2	0	0
Specificity	90.5%	100%	100%
Sensitivity	50%	50%	100%

GM01750/9	Yes	Yes	Yes
GM01750/14	Yes	Yes	Yes
Number of false positives	1	0	0
Specificity	95.2%	100%	100%
Sensitivity	100%	100%	100%

GM03134/8	Yes	Yes	Yes
Number of false positives	3	1	3
Specificity	86.4%	95.5%	97.9%
Sensitivity	100%	100%	100%

GM03563/3	Yes	Yes	Yes
GM03563/9	No	No	Yes
Number of false positives	8	5	0
Specificity	61.9%	76.2%	100%
Sensitivity	50%	50%	100%

GM05296/10	Yes	Yes	Yes
GM05296/11	Yes	Yes	Yes
Number of false positives	3	0	2
Specificity	88%	100%	99.3%
Sensitivity	100%	100%	100%

GM07081/7	Yes	Yes	Yes
GM07081/15	No	No	No
Number of false positives	1	0	0
Specificity	95.2%	100%	100%
Sensitivity	50%	50%	100%

GM13031/17	Yes	Yes	Yes
Number of false positives	5	3	1
Specificity	79.2%	87.5%	98.8%
Sensitivity	100%	100%	100%

GM13330/1	Yes	Yes	Yes
GM13330/4	Yes	Yes	Yes
Number of false positives	8	5	0
Specificity	61.9%	76.2%	100%
Sensitivity	100%	100%	100%

From Table 4, it is evident that the new Bayesian-CPCM approach can detect the CNV regions with highest specificities and sensitivities. The false positives of the Bayesian-CPCM on two of the chromosomes are due to outliers and noise in the original data.

It is worth noting that the CNV or aberration regions in these 9 fibroblast cell lines that were found using our proposed Bayesian-CPCM approach are also consistent with those identified in Olshen et al. [4], Chen and Wang [19], Venkatraman and Olshen [24]. However, our new approach, Bayesian-CPCM, neither involve heavy computations as that of CBS algorithm in Olshen et al. [4], nor any asymptotic distribution as required in our earlier work [19].

## 4. Conclusion

A Bayesian approach for identifying CNVs in aCGH profile modeled by a compound Poisson process is proposed in this paper. Theoretical results of the Bayesian analysis are obtained and the algorithm has been implemented with Matlab. Applications of the proposed method to several aCGH data sets have demonstrated its effectiveness. Extensive simulation results indicate that the proposed method can work effectively for various cases. The most distinct feature of the proposed Bayesian-CPCM approach, when compared with existing methods in the literature, is its use of both biomarker positions (hence distances) and the log-intensity ratio information in the model. Another important aspect of the proposed approach is that it characterizes the posterior probability of the loci being a CNV. With the common knowledge of probability, the users can easily judge if there is a CNV at a locus by using the posterior probability together with their biological knowledge.

There are many computational and statistical approaches now available for analyzing aCGH data in the literature. But those approaches, especially the CBS of Olshen et al. [4] and MVCM of Chen and Wang [19], are all targeted on modeling the log ratio in aCGH data. In this paper, we have used a new approach to model both the biomarker position and the log ratio intensity in aCGH data. In other words, the most distinct feature of the proposed Bayesian-CPCM approach, among other existing methods, is the use of both biomarker position information (hence distances) and the log-intensity ratios in the model. The size of the sliding window is very important in search multiple change points in a whole sequence. The criterion of choosing the optimal window size remains to be done in the future.
